# Correlation between the CD8^+^ T cell/IFN-*γ* axis and clinical disease progression at different phases of ITP

**DOI:** 10.3389/fped.2026.1905260

**Published:** 2026-07-31

**Authors:** Jie Ding, Chentao Shen, Jianmei Chen, Ziyang Cheng, Jianqin Li

**Affiliations:** 1 Department of Hematology and Oncology, Children’s Hospital of Soochow University, Suzhou, China; 2 Suzhou Wujiang District Children’s Hospital, Suzhou, China; 3 Sir Run Run Hospital, Nanjing Medical University, Nanjing, China

**Keywords:** CD8^+^ T cells, childhood ITP, immune characteristics, interferon-*γ*, therapeutic response

## Abstract

**Background:**

Primary immune thrombocytopenia (ITP) represents the most common acquired bleeding disorder among children. Since its immune mechanism is not fully clarified, precise immune markers still need to be identified for guiding and intervening in recurrent and refractory cases.

**Methods:**

This cross-sectional study included children diagnosed with ITP between August 2022 and November 2024. Peripheral blood levels of CD3^+^ T, CD4^+^ T, CD8^+^ T, and B cells were quantified via flow cytometry analysis. Intracellular IFN-*γ* expression was analyzed. Spearman correlation analysis was conducted in combination with platelet count and WHO bleeding score.

**Results:**

Compared with healthy controls, children with ITP exhibited a markedly reduced platelet count and elevated bleeding frequency (both *P* *<* *0.*0001). The proportion of CD8^+^ T cells significantly increased in both the relapse group and the severe group, and the CD4^+^/CD8^+^ ratio decreased in the severe group. The proportions of CD3^−^IFN-*γ* cells, CD4^+^ IFN-*γ*T cells, and CD8^+^ IFN-*γ*T cells were all higher in ITP children than in healthy controls, with higher CD3^+^ CD8^+^ IFN-*γ*T cells expression detected in the severe group (all *P* < 0.05). Platelet was negatively correlated with the proportion of CD3^+^ CD8^+^ IFN-*γ*T cells (r = −0.2583, *P* = 0.012).

**Conclusion:**

Aberrant activation of the CD8^+^ T cell/IFN-*γ* axis is closely associated with thrombocytopenia, disease recurrence, and severity in pediatric ITP, and may serve as a potential immune-targeted intervention candidate for refractory ITP.

## 1 Introduction

Immune thrombocytopenia (ITP) represents the most prevalent acquired hemorrhagic disorder in pediatric populations, characterized by a peripheral blood plate count of less than 100 × 10⁹/L, accompanied by bleeding risk of skin, mucous membrane, or visceral (1, 2). Although about 80% of cases present with an acute self-limiting course, 20%–30% progress to chronic ITP, resulting in a long-term treatment burden and reduced life quality (3, 4). According to global epidemiological statistics, childhood ITP has an annual incidence of approximately 4–5 cases per 100,000 individuals, and the disease exhibits marked clinical heterogeneity, with clinical presentations ranging from asymptomatic thrombocytopenia to life-threatening bleeding events (5, 6). These differences imply the existence of distinct immunological subtypes of ITP, highlighting the urgent need for stratified management guided by molecular markers.

Conventionally, the pathogenesis of ITP is considered to depend on autoantibody-mediated platelet destruction, whereas recent investigations have revealed complex immune dysregulation including T-cell dysfunction, cytokine imbalance, immune regulatory defects, and microbiota-associated immune modulation (7, 8). In particular, CD8⁺ cytotoxic T cells (CTLs) directly attack platelets and megakaryocytes via the perforin-granzyme pathway, and the disordered network of pro- and anti-inflammatory factors resulting from imbalances among Th1/Th2/Th17/Treg cell subsets further aggravates immune tolerance deficiency (9, 10). Interferon -*γ* (IFN-*γ*), a pivotal effector molecule of the Th1-type immune response, not only activates macrophages to facilitate platelet phagocytosis but also enhances the cytotoxicity of CTLs by up-regulating MHC-I expression (11, 12). However, a research gap still exists regarding the dynamic changes of CD8^+^ T cells and the IFN-*γ* axis in pediatric ITP and their clinical associations.

The current first-line therapies for ITP, including glucocorticoids and intravenous immunoglobulin (IVIG), can transiently elevate platelet counts, but about 25 percent of pediatric patients exhibit treatment resistance or relapse (13, 14). Clinical observations have demonstrated that variations in treatment response may be associated with specific immune profiles: for instance, pediatric patients with elevated IFN-*γ* levels show a poorer response to IVIG, while restoration of Treg cell frequencies is positively correlated with long-term disease remission (15, 16). However, the majority of existing studies have centered predominantly on adult ITP, and immune developmental dynamics unique to the pediatric population may influence both disease phenotype and treatment response. Systematic research on the CD8⁺ T cell/IFN-*γ* axis remains particularly scarce with respect to disease stage (newly diagnosed/persistent/chronic), bleeding risk, and treatment response.

This study employed multi-parameter flow cytometry to systematically characterize peripheral blood immune cell subsets (involving CD3^+^ T, CD4⁺ T, CD8⁺ T, and B cells) and intracellular IFN-*γ* expression in pediatric ITP patients, assess their correlations with clinical parameters (i.e., platelet count and bleeding score), and explore the value of the CD8⁺ T cell/IFN-*γ* axis in the therapeutic response.

## 2 Materials and methods

### 2.1 Participants

A total of 95 children diagnosed with ITP by clinical and laboratory tests who were admitted to the outpatient and inpatient departments of Soochow University Children's Hospital from August 2022 to November 2024 were included in this study. The diagnostic criteria were in accordance with the 2019 edition of the “Diagnosis and Treatment Guidelines for Primary Immune Thrombocytopenia in Children” by the Hematology Group of the Pediatrics Branch of the Chinese Medical Association. The exclusion criteria were as follows: a diagnosis of secondary thrombocytopenia due to thyroid disorders, autoimmune diseases, drug induction, or alloimmunity; lymphoproliferative disorders; myelodysplastic conditions, including aplastic anemia and myelodysplastic syndrome; chronic liver disease with hypersplenism; malignant hematological diseases; infections; pseudothrombocytopenia; microvascular hemolytic anemia (including acquired and hereditary thrombotic thrombocytopenic purpura; and hereditary thrombocytopenia.

Besides, a total of 38 healthy children who received routine physical examinations at the pediatric health outpatient unit at the same time were selected as health controls in this study. They had no history of thrombocytopenia, normal platelet test results, no immune disease, no family history of immune disease, and no treatment with immunosuppressants, glucocorticoids, or biologics.

All participants’ peripheral blood cellular immune levels and IFN-*γ* expression levels were detected before initial treatment. ITP patients were categorized into newly diagnosed, persistent, chronic, and severe subgroups according to their clinical stage. Specifically, newly diagnosed ITP subgroup included children who have been diagnosed within 3 months; persistent ITP subgroup included children with persistent thrombocytopenia for 3–12 months after diagnosis, including those who have not achieved spontaneous remission or cannot maintain complete remission after discontinuation of treatment; chronic ITP subgroup included children who have experienced a decrease in platelets for more than 12 months; severe ITP subgroup included children with platelet count <10 × 10^9^L, and bleeding symptoms that require treatment at the time of visit or new bleeding symptoms occur during conventional treatment, and need other platelet-raising drugs for treatment or the dosage of existing drugs to be increased. Additionally, ITP patients were stratified into an initial diagnosis group and a recurrence group based on relapse status at diagnosis. Detailed parameters between ITP patients and healthy controls (HC) are shown in Supplementary Table S1. We conducted this study in compliance with the principles outlined in the Declaration of Helsinki, and written informed consent was acquired from every enrolled patient. Additionally, the ethical review board of Soochow University Children's Hospital granted official approval for this research project (Ethics Number: 2024CS016).

### 2.2 Sample processing

Peripheral venous blood (2–4 mL, anticoagulated with EDTA) was obtained from children in the ITP patient group and HC group at enrollment. Samples were immediately stored at 4 °C, and plasma and blood cells were isolated within 4 h. The separated plasma was aliquoted into microcentrifuge tubes, labeled and coded, then preserved at −80 °C pending subsequent testing. Meanwhile, peripheral blood mononuclear cells (PBMCs) were extracted via density gradient centrifugation for flow cytometric analysis. The proportion of peripheral blood CD3⁺ T, CD4⁺ T, CD8⁺ T lymphocytes, B cells, as well as the CD4⁺/CD8⁺ ratio was determined. Following stimulation of PBMCs with anti-CD3 monoclonal antibody, the expression of IFN-*γ* in CD3⁻, CD3⁺CD8⁺ T cells, and CD3⁺CD4⁺ T cells was analyzed by flow cytometry.

### 2.3 Experimental methods

#### 2.3.1 Staining of peripheral blood immune cell subsets

Peripheral blood mononuclear cells (PBMCs) were extracted, diluted with flow cytometry staining buffer, and adjusted to a cell density of 1 × 10^6^ cells per milliliter. Cells were washed with 300 μL of F-PBS, centrifuged at 4,000 rpm for 3 min, and incubated with fluorescently labeled antibodies against CD3, CD19, and CD4 in the dark at 4 °C for 30 min. After incubation, cells were rinsed using 400 μL F-PBS and centrifuged at 4,000 rpm for 3 min, with the subsequent supernatant removed. The cell pellets were reconstituted in 200 μL F-PBS, and flow cytometry was applied to detect the expression levels of surface molecular markers on cells.

#### 2.3.2 Flow cytometry detection of IFN-*γ* expression in T cell subsets

The isolated PBMCs were resuspended in medium and adjusted to a density of 1 × 10^6^ cells/mL, followed by the addition of anti-CD3 monoclonal antibody (to stimulate the TCR-CD3 complex) and the Golgi blocker monomycin. Cells were then incubated at 37 °C in a 5% CO_2_ atmosphere for 4–6 h. Subsequently, cells were washed with 300 μL of F-PBS (centrifuged at 4,000 rpm for 3 min) and stained with fluorescently labeled surface antibodies, including anti-CD3 (Brilliant Violet 510™, clone UCHT1), anti-CD4 (FITC, clone RPA-T4), and anti-CD8 (APC/Cyanine7, clone SK1) (all from BioLegend, USA). The cells were incubated in the dark at 4 °C for 30 min. After washing, cells were fixed with 200 μL of intracellular fixation buffer at room temperature in the dark for 20 min, permeabilized with 500 μL of permeabilization buffer (centrifuged at 8,000 rpm for 3 min), and incubated with an intracellular antibody against IFN-*γ* (Brilliant Violet 421™, clone B27, BioLegend) at 4 °C for 1 h. Finally, the cells were washed and resuspended in 200 μL of permeabilization buffer and examined by flow cytometry. The proportion and expression level of IFN-*γ*-positive cells were determined using FlowJo software. To identify specific immune cell subsets, a sequential gating strategy was applied: first, lymphocytes were gated based on forward scatter (FSC-A) and side scatter (SSC-A) characteristics. Next, singlet cells were selected by gating on FSC-A versus SSC-H to exclude doublets. Within the singlet lymphocyte population, T cells and B cells were identified by gating on CD3^+^ and CD19^+^ events, respectively. Within the singlet lymphocyte population, T cells were identified by gating on CD3^+^ events. The CD3^+^ T cells were subsequently analyzed for CD4 and CD8 expression to directly delineate the CD3^+^ CD4^+^ and CD3^+^ CD8^+^ subpopulations. Finally, the proportion and expression level of IFN-*γ* within each specific T-cell subset were quantified.

### 2.4 Statistical analysis

Categorical data were expressed as percentages, and the chi-square test was adopted for between-group comparisons. Continuous variables conforming to a normal distribution were presented as mean ± standard deviation. The differences between the two separate groups were assessed via the independent-sample t-test. For the comparison of data across three or more groups, one-way ANOVA was adopted initially. The Tukey test was used for homogeneity of variance, and the Dunnett T3 test was employed for non-homogeneity of variance. Data with a non-normal distribution were expressed as median (M) along with interquartile range (P25, P75). The Mann–Whitney U test was adopted for two-group analyses, while the Kruskal–Wallis test was applied in comparisons involving three or more groups. Pairwise multiple comparisons were further conducted using the Dunnett method, where overall significance was detected. Spearman's correlation coefficient was adopted for correlation analysis, and a *p*-value <0.05 was defined as the threshold for statistical significance. Statistical analysis and graphical visualization of the experimental data were conducted using GraphPad Prism 8 software.

## 3 Results

### 3.1 Clinical characteristics of participants

**Figure 1:**
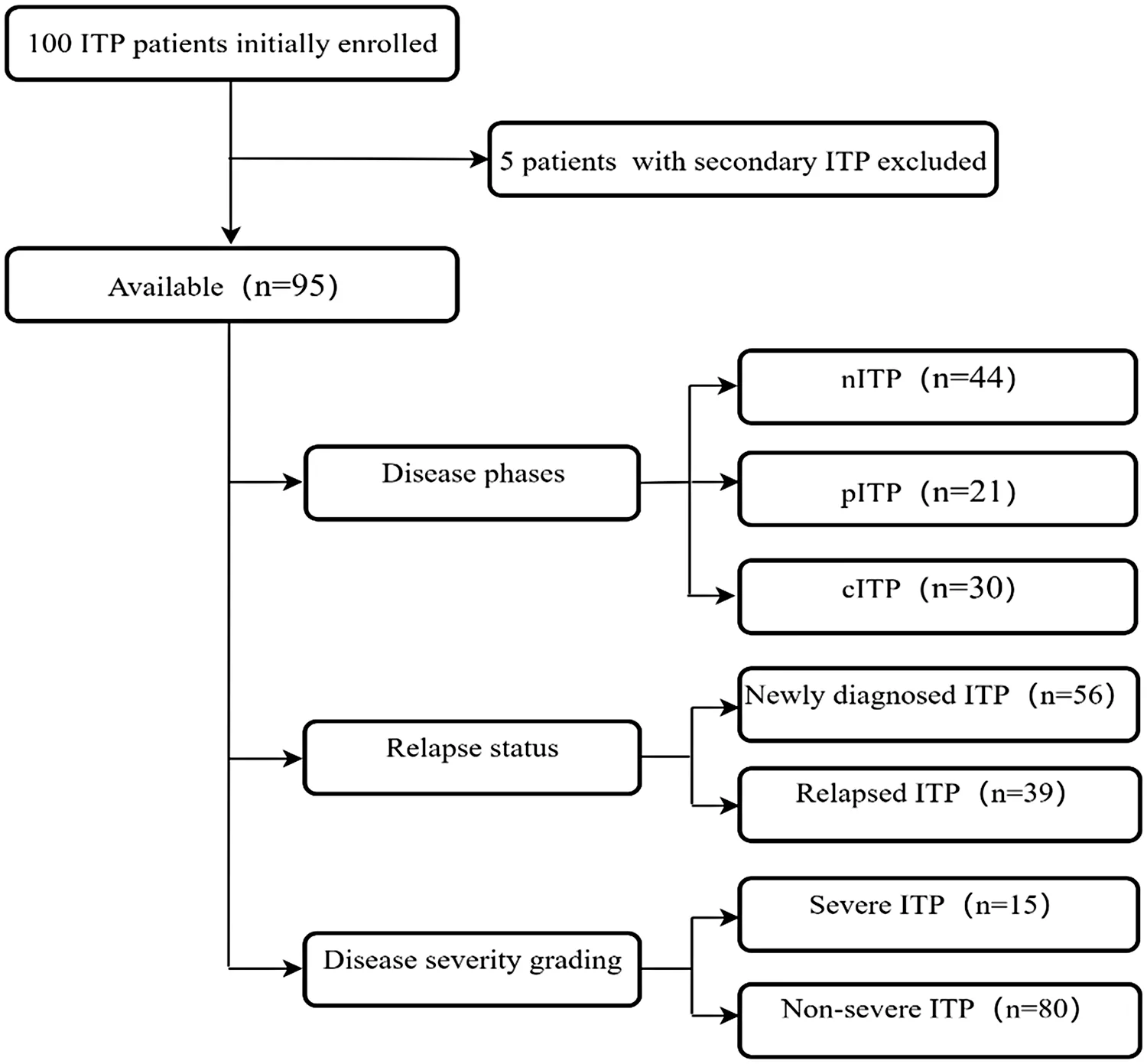
Screening procedure for patient data starts with the preliminary enrollment of study subjects.

The flowchart of participant screening and categorization is shown in Figure 1. Comparison of baseline characteristics of different ITP groups and HC groups is shown in Table 1. Platelet counts in the ITP group were significantly lower than those in the control group, and the incidence of bleeding was higher (all *P* < 0.0001). The two groups showed no statistically distinct variations in terms of age and gender distribution. The incidence of bleeding in the nITP group was significantly higher than that in the pITP group (65.9% vs. 16.7%), and the platelet level in the cITP group was significantly lower than that in the pITP group (22.5 × 10^9^/L vs. 52.0 × 10^9^/L). The nITP group had a markedly lower median age compared with the cITP group (56.5 months vs. 89.2 months), while no notable discrepancy in gender composition was observed across the three ITP subgroups (*P* = 0.346). Additionally, the incidence of bleeding in the severe ITP group was higher than that in the non-severe group (80.0% vs. 37.5%), and platelet count was lower (5.0 × 10^9^/L vs 30.5 × 10^9^/L), with no difference in age or gender between the two groups (*P* = 0.385 and 0.870).

**Table 1:** Basic information of children with ITP.

Items	Relapse status				Disease phases				Disease severity grading		
	Newly diagnosed ITP (*n* = 56)	Relapsed ITP (*n* = 39)	Healthy controls (*n* = 38)	*P*	nITP (*n* = 44)	pITP (*n* = 21)	cITP (*n* = 30)	*P*	Severe ITP (*n* = 15)	Non-severe ITP (*n* = 80)	*P*
Age (months)	66.00 (28.00, 96.00)	62.00 (32.00, 100.00)	81.00 (60.00, 99.00)	0.502	34.00 (13.25, 97.50) &	66.00 (39.50, 89.00)	89.00 (61.50, 120.3)	0.007	59.93 ± 38.97	70.89 ± 45.49	0.385
Gender (male/female)	25/31	18/21	20/18	0.736	23/21	7/14	13/17	0.346	7/8	36/44	0.870
Platelet, ×10^9^/L	25.50 (15.25, 50.25)^a^	30.00 (14.00, 67.00)^a^	310.50 (262.80, 337.00)	<0.0001	25.50 (12.75, 51.75)	52.00 (21.00, 65.50) &	22.50 (13.75, 34.25)	0.048	5.00 (3.00, 7.00) @	30.50 (20.50, 59.75)	<0.0001
Hemorrhagic symptoms	29/56^a^	12/39^a^	0/38	<0.0001	29/44&#	3/18	9/30	<0.0001	12/15@	30/80	0.029

a Compared with the HC group, *P* *<* 0.05. &Compared with the cITP group, *P* < 0.05*.* #Compared with pITP group, *P* < 0.05. @Compared with the non-severe ITP group, *P* < 0.05.

### 3.2 Comparison of the differential expression of immune cells between ITP children and HC

**Figure 2:**
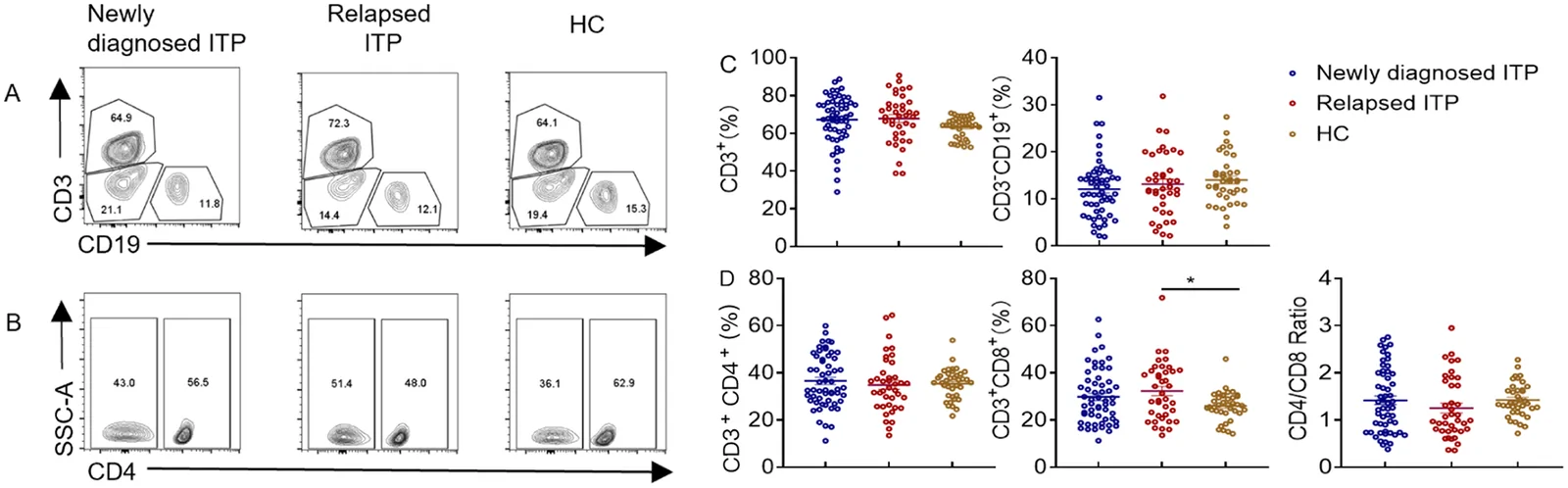
Immune cell subsets levels in the newly diagnosed ITP, relapsed ITP and HC. A: Percentages of CD3^+^ T cells and CD3^−^CD19^+^ cells in total lymphocytes in the ITP initial diagnosis group (*n* = 56), recurrence group (*n* = 39), and HC group (*n* = 38); B: Representative flow cytometry plots of CD4^+^ T cells and CD8^+^ surrogate T cells gated within the parent CD3^+^ T cell population across the three groups. The CD3^+^ CD4^-^ subset was mathematically quantified as the surrogate proxy for CD8^+^ T cells; C: Three groups of CD3^+^ T cells and the percentage of CD3^−^CD19^+^ cells in total lymphocytes; D: Percentages of CD4^+^ T cells and CD8^+^ T cells in total lymphocytes and the ratio of CD4% to CD8% in the three groups. *P *<* 0.05.

**Figure 3:**
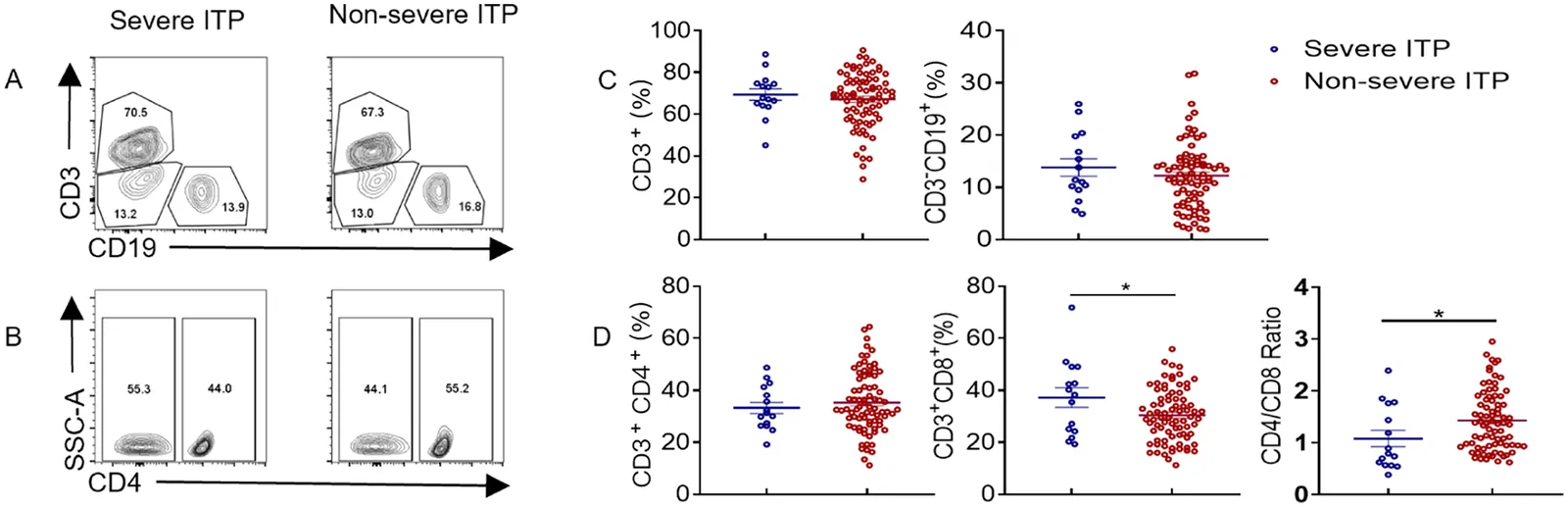
Immune cell subsets levels in severe ITP and non-severe ITP. A: Percentages of CD3^+^ T cells and CD3^−^CD19^+^ cells in total lymphocytes in the peripheral blood of the severe ITP group (*n* = 15) and the non-severe ITP group (*n* = 80); B: Percentages of CD4^+^ T cells and CD8^+^ T cells in the upper phylum of CD3^+^ T cells in both groups. The CD3^+^ CD4^-^ subset was mathematically quantified as the surrogate proxy for CD8+ T cells; C: Percentages of CD3^+^ T cells and CD3^−^CD19^+^ cells in total lymphocytes in both groups; D: Percentages of CD4^+^ T cells and CD8^+^ T cells in total lymphocytes and the ratio of CD4% to CD8% in both groups. *P *<* 0.05.

The proportion of CD8^+^ T cells in the relapsed ITP group (40.22%) was significantly higher than that in the HC group (23.20%) and the newly diagnosed ITP group (27.86%). No statistically significant differences in the expression levels of CD3⁺ T cells, CD4⁺ T cells, B cells, and the CD4^+^/CD8^+^ ratio were observed among the three groups (Figure 2 and Table 2). The severe subgroup exhibited elevated CD8^+^ T cell levels and a decreased CD4^+^/CD8^+^ ratio in comparison with the non-severe group. There were no significant differences in the expression of CD3⁺ T cells, CD4⁺ T cells, and B cells between the two groups (Figure 3).

**Table 2:** The level of immune cell subpopulations before treatment in the newly diagnosed ITP, relapsed ITP, HC group and severe group vs. non-severe group.

Items	Relapse status			*P*	Disease severity grading		*P*
	Newly diagnosed ITP (*n* = 56)	Relapsed ITP (*n* = 39)	HC (*n* = 38)		Severe ITP (*n* = 15)	Non-severe ITP (*n* = 80)	
CD3%^a^	68.00 (61.05, 76.30)	66.40 (56.55, 74.30)	65.60 (59.88, 69.20)	0.228	67.80 (64.20, 74.80)	69.05 (59.13, 76.48)	0.707
CD4%^a^	32.57 (26.54, 41.10)	35.20 (27.32, 40.80)	34.42 (28.43, 39.09)	0.739	31.27 (26.43, 41.28)	32.75 (27.20, 45.24)	0.549
CD8%^a^	27.86 (22.56, 36.37)**###	40.22 (29.21, 49.10)****	23.20 (16.23, 29.68)	<0.0001	42.41 (28.16, 50.97)***	28.82 (21.00, 36.76)	0.0009
B cell %^a^	13.75 (9.61, 16.70)	12.10 (7.82, 19.40)	11.90 (8.27, 14.45)	0.156	11.40 (9.50, 19.82)	12.00 (7.21, 15.10)	0.477
CD4/CD8 ratio	1.09 (0.67, 1.80)	1.13 (0.81, 1.82)	1.31 (0.92, 1.63)	0.414	0.84 (0.62, 1.07)**	1.20 (0.88, 1.73)	0.005

a refers to the percentage of total lymphocytes; ***P *<* 0.001 compared with HC group, ****P < 0.0001 compared with HC group; ###*P* < 0.001 com*p*ared with the initial diagnosis group.

**Figure 4:**
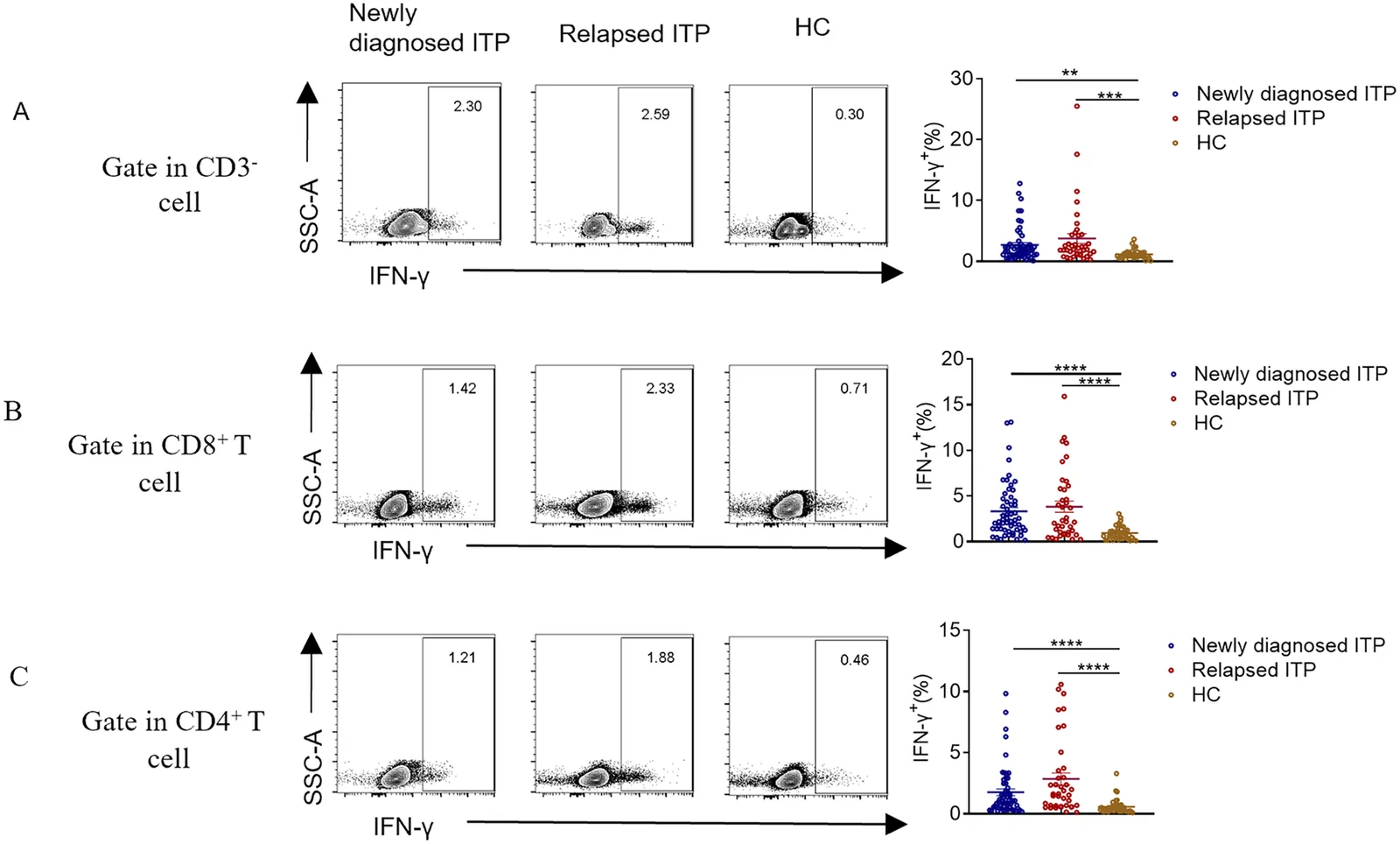
IFN-*γ* levels in PBMCs of newly diagnosed ITP, relapsed ITP, and HC groups. Analysis of IFN-*γ* cell expression after *in vitro* stimulation of PBMCs with anti-CD3 antibodies. A: Percentage of IFN-*γ* cells in peripheral blood to CD3^−^ cells in the ITP initial diagnosis group, recurrence group and HC group; B: Percentage of IFN-*γ* cells in peripheral blood to CD3^+^ CD8^+^ T cells in the ITP initial diagnosis group, recurrence group and HC group; C: Percentage of IFN-*γ* cells in peripheral blood to CD3^+^ CD4^+^ T cells in the ITP initial diagnosis group, recurrence group and HC group. **P < 0.01; ***P < 0.001; ****P < 0.0001.

**Figure 5:**
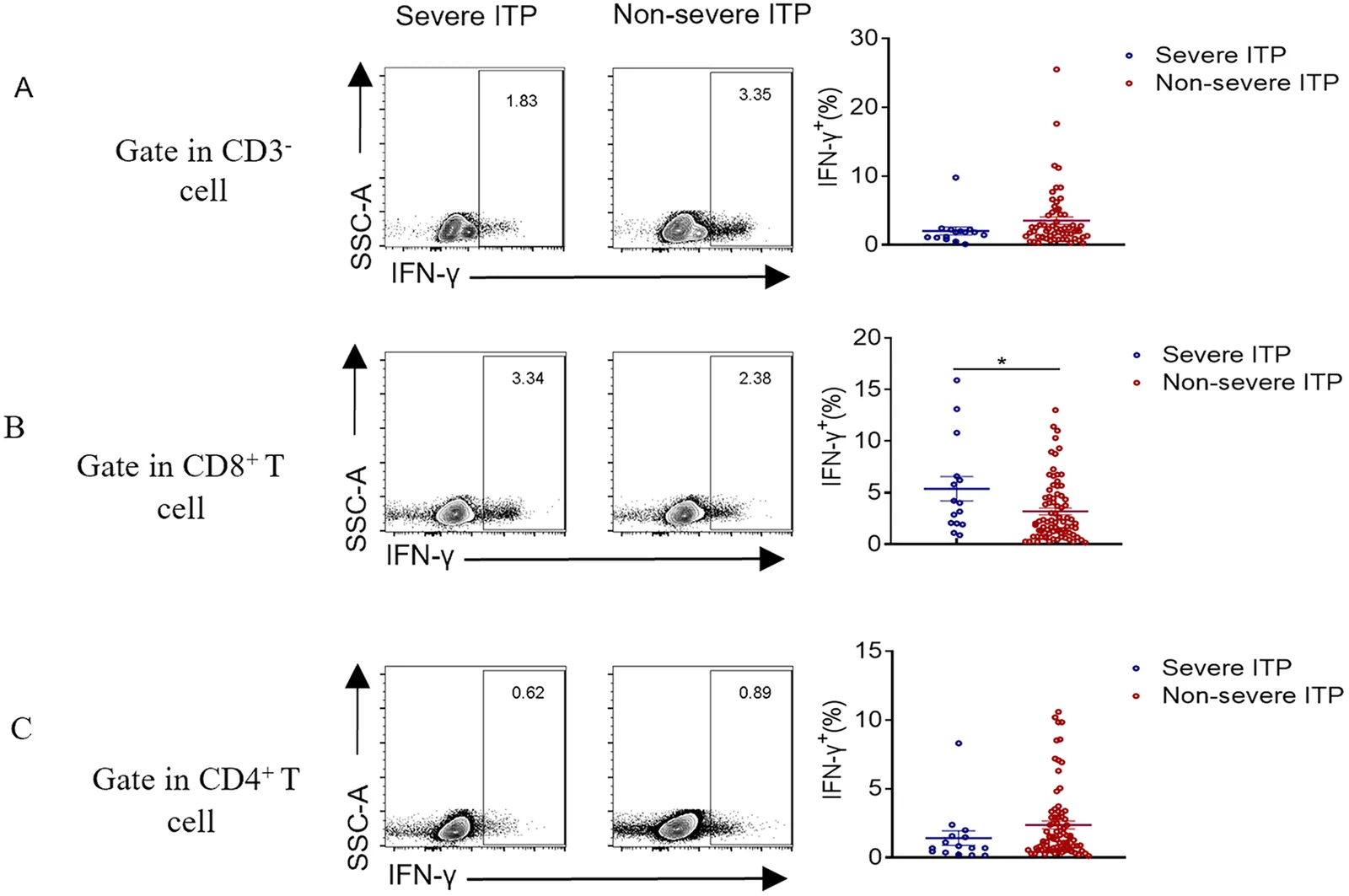
IFN-*γ* levels in PBMCs of severe ITP and non-severe ITP groups. A: Percentage of IFN-*γ* cells in peripheral blood to CD3^−^ cells in both severe ITP and non-severe ITP groups; B: Percentage of IFN-*γ* cells in peripheral blood to CD3^+^ CD8^+^ T cells in the severe ITP and non-severe ITP groups; C: Percentage of IFN-*γ* cells in peripheral blood to CD3^+^ CD4^+^ T cells in the severe ITP and non-severe ITP groups. *P *<* 0.05.

Flow cytometry results showed that the proportions of the above three types of IFN-*γ* positive cells in newly diagnosed and relapsed ITP patients significantly increased compared with HC (all *P* < 0.0001), while there was no statistically significant difference between the newly diagnosed group and the relapsed group (*P* > 0.05) (Figure 4 and Table 3). Mann–Whitney U test analysis showed that the expression of CD3^+^ CD8^+^ IFN-*γ* T cells in children with severe ITP was higher than that in the non-severe group, and there was no significant difference in the expression of CD3^−^IFN-*γ* cells and CD3^+^ CD4^+^ IFN-*γ* T cells between the two groups (*P* = 0.172, *P* = 0.071) (Figure 5).

**Table 3:** The expression of IFN-*γ* in CD3^−^, CD4^+^ T, CD8^+^ T cells in the peripheral blood of the diagnosis group, recurrent group, HC group, and severe and non-severe ITP group.

Items	Relapse status			*P*	Disease severity grading		*P*
	New diagnosed ITP (*n* = 56)	Relapsed ITP (*n* = 39)	HC (*n* = 38)		Severe ITP (*n* = 15)	Non-severe ITP (*n* = 80)	
CD3^−^IFN-*γ*^+^ (%)	1.79 (1.03, 2.82)**	1.99 (1.22, 4.38)***	0.97 (0.77, 1.44)	<0.0001	1.83 (1.05, 2.20)	2.01 (1.09, 4.28)	0.172
CD3^+^ CD8^+^ IFN-γ^+^ (%)	2.31 (1.29, 4.64)****	2.18 (0.89, 5.81)****	0.76 (0.38, 1.31)	<0.0001	4.03 (2.03, 6.60)#	2.16 (1.03, 4.61)	0.034
CD3^+^ CD4^+^ IFN-γ^+^ (%)	1.05 (0.48, 2.39)****	1.62 (0.67, 3.53)****	0.37 (0.20, 0.68)	<0.0001	0.69 (0.37, 1.57)	1.34 (0.59, 2.93)	0.071

Compared with HC group, **P *<* 0.01, ***P < 0.001, ****P < 0.0001; #*P* < 0.05 compare*d* with the non-severe ITP group.

The immune characteristics of children with different nITP, pITP, and cITP were analyzed, and the results showed that among the three study groups, no obvious statistical disparities were observed in the proportion of CD3⁺, CD4⁺ T, CD8⁺ T, and B cells, as well as the CD4⁺/CD8⁺ ratio. (all *P* *>* 0.05); There were no significant differences in the expression levels of CD3⁻IFN-*γ*⁺ cells, CD3⁺ CD8⁺ IFN-*γ*⁺ T cells, and CD3⁺CD4⁺IFN-*γ*⁺ T cells among the three groups (Table 4).

**Table 4:** nITP, pITP, cITP patient's pre-treatment levels of immune cell subsets and IFN-γ levels.

Items	nITP (*n* = 44)	pITP (*n* = 21)	cITP (*n* = 30)	*P*
CD3% within lymphocyte gate	67.90 (60.93, 74.95)	74.50 (61.45, 80.60)	68.05 (56.60, 76.60)	0.380
CD4% within lymphocyte gate	32.64 (29.41, 41.36)	35.20 (29.25, 46.96)	36.60 (25.58, 49.12)	0.860
CD8% within lymphocyte gate	29.47 (22.71, 38.83)	23.29 (17.20, 34.36)	32.65 (21.42, 42.91)	0.083
B cell % within lymphocyte gate	12.60 (7.65, 15.33)	11.50 (7.38, 15.45)	11.85 (7.27, 15.78)	0.898
CD4/CD8 ratio	1.12 (0.77, 1.67)	1.71 (0.83, 2.28)	1.03 (0.67, 1.90	0.363
CD3^−^IFN-γ^+^ (%)	2.01 (0.95, 3.88)	1.96 (1.11, 3.13)	1.79 (1.26, 3.25)	0.867
CD3^+^ CD8^+^ IFN-γ^+^ (%)	2.05 (1.11, 4.74)	2.14 (1.45, 4.49)	3.64 (0.85, 5.72)	0.616
CD3^+^ CD4^+^ IFN-γ^+^ (%)	1.13 (0.65, 3.38)	0.86 (0.56, 2.35)	1.57 (0.55, 2.36)	0.549

### 3.3 Correlation analysis of immune characteristics with clinical parameters of ITP

**Figure 6:**
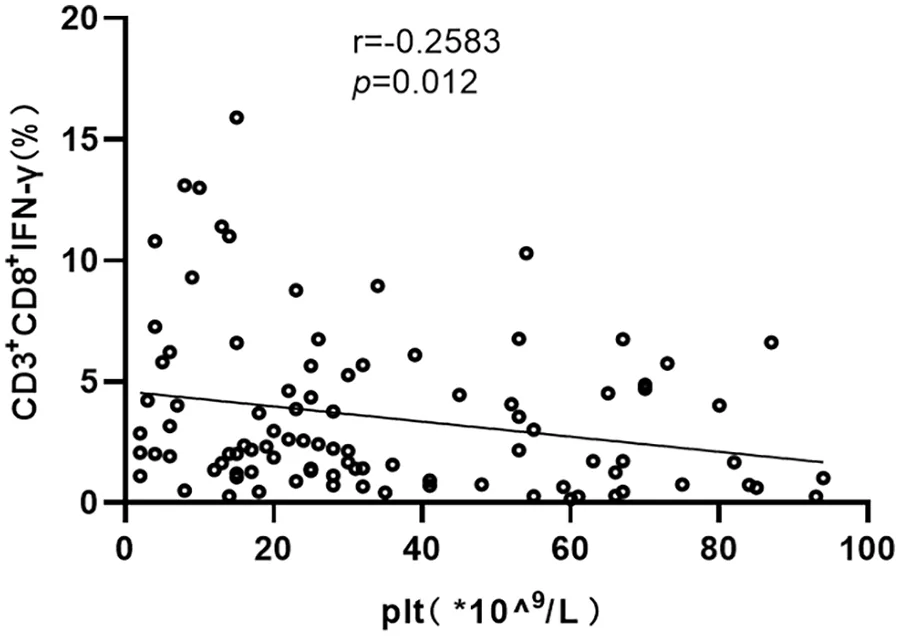
Analysis of the correlation between peripheral blood CD3 ^+^ CD8 ^+^ IFN-*γ*^+^T cells and platelet counts in patients with ITP.

The Spearman correlation analysis revealed that, in children diagnosed with ITP, the proportions of peripheral blood CD3⁺, CD4⁺ T, CD8⁺ T, and B cells, as well as the CD4⁺/CD8⁺ ratio, exhibited no statistically significant correlation with platelet counts or bleeding manifestations (all *P* *>* 0.05). Further analysis revealed that platelet counts were only negatively correlated with CD3^+^ CD8^+^ IFN-*γ*^+^ T cells (r = −0.2583, *P* = 0.012), and not correlated with CD3^−^IFN-*γ*^+^ cells or CD3^+^ CD4^+^ IFN-*γ*^+^ T cells; None of the subpopulations of IFN-*γ* positive cells were associated with bleeding symptoms (Figure 6 and Table 5).

**Table 5:** Correlation analysis between the peripheral blood IFN-γ cell expression level, the percentage of immune cell subsets and the platelet, bleeding symptoms children with ITP.

Items	Platelets		Hemorrhagic symptoms	
	Correlation coefficient	*P*	Correlation coefficient	*P*
CD3^−^IFN-γ^+^ (%)	0.062	0.552	0.002	0.979
CD3^+^ CD8^+^ IFN-γ^+^ (%)	−0.258	0.012	−0.039	0.701
CD3^+^ CD4^+^ IFN-γ^+^ (%)	−0.170	0.101	−0.061	0.558
CD3% within lymphocyte gate	−0.002	0.988	−0.137	0.185
CD4% within lymphocyte gate	−0.049	0.635	−0.040	0.703
CD8% within lymphocyte gate	0.118	0.255	−0.054	0.603
B cell % within lymphocyte gate	−0.191	0.064	0.083	0.424
CD4/CD8 ratio	−0.136	0.188	0.001	0.994

## 4 Discussion

The present study focused on the CD8^+^ T cell/IFN-*γ* axis and systematically characterized its expression profiles across distinct clinical states of childhood ITP, including initial diagnosis, disease recurrence, varying disease courses, and severe vs. non-severe disease subtypes, as well as its association with routine clinical indicators. These findings provided critical experimental evidence for elucidating the underlying pathogenesis of childhood ITP, identifying potential diagnostic and prognostic biomarkers, and optimizing clinical treatment strategies for this pediatric autoimmune disorder.

Our results demonstrated that the proportion of CD8^+^ T cells was significantly elevated in the recurrent ITP group compared with both the newly diagnosed ITP group and the HC group. Additionally, CD8^+^ T cell expression was notably higher in the severe ITP group relative to the non-severe group, accompanied by a disrupted CD4^+^/CD8^+^ ratio. Notably, there were no significant intergroup differences observed in the proportions of CD3^+^ T cells, CD4^+^ T cells, CD8^+^ T cells, or B cells. These findings aligned with the core consensus of recent research on the immunopathogenesis of ITP, which posits that CD8^+^ T cells, as pivotal effector immune cells, contribute to immune-mediated platelet destruction via aberrant activation (17–19). Studies have validated that CD8^+^ T cells mediate platelet depletion through two primary mechanisms: direct cytotoxicity triggered by recognition of platelet surface autoantigens, or indirect acceleration of platelet apoptosis via secretion of pro-inflammatory cytokines that foster a pathogenic pro-inflammatory microenvironment (20, 21). The current study further refines this understanding by demonstrating that abnormal CD8^+^ T cell activation is not only a common immune feature of childhood ITP but also closely related to disease recurrence and clinical severity, suggesting that this T cell subset may serve as a core driver of disease progression and a potential contributor to poor treatment responsiveness and disease recurrence (22–25). Compared with newly diagnosed cases, pediatric patients with recurrent ITP have enhanced CD8^+^ T cell activation, which may reflect the accumulation of immune memory cells or persistent dysregulation of the immune regulatory network during disease progression, providing a potential marker for risk stratification of disease recurrence. Furthermore, the elevated CD8^+^ T cell expression and reduced CD4^+^/CD8^+^ ratio in severe ITP cases further corroborate the role of immune imbalance in disease exacerbation, providing a reference for early warning of critical illness and targeted stratified clinical intervention. However, due to the relatively small sample size of the severe ITP group, large-scale prospective studies should be conducted in the future to further verify the potential biomarker applications and therapeutic implications of CD8^+^ T cells.

IFN-*γ*, a classic Th1-type pro-inflammatory cytokine, exerts a key regulatory role in immune dysregulation underlying autoimmune diseases. It mediates immunopathological damage by enhancing antigen-presenting cell function, promoting the recruitment of inflammatory effector cells, activating cytotoxic T lymphocytes, and amplifying aberrant immune responses (26, 27). In the present study, *in vitro* stimulation experiments confirmed that the proportions of CD3^−^IFN-*γ*^+^ cells, CD3^+^ CD4^+^ IFN-*γ*^+^ T cells, and CD3^+^ CD8^+^ IFN-*γ*^+^ T cells were all significantly higher in both newly diagnosed and recurrent childhood ITP patients than in HC. Notably, only the expression of CD3^+^ CD8^+^ IFN-*γ*^+^ T cells was associated with the severity of the disease. This result revealed two core pathological characteristics of childhood ITP: first, IFN-*γ* -associated immune activation is an inherent pathological feature of childhood ITP, independent of initial diagnosis or disease recurrence status, suggesting that abnormal activation of this inflammatory pathway may persist throughout the entire disease course rather than occurring as a secondary response to immune stress following disease recurrence; second, CD8^+^ T cells represent the primary effector cell subset responsible for IFN-*γ* secretion in this context, and IFN-*γ* derived from these cells may act as a core molecular mediator of thrombocytopenia. Unlike CD4^+^ IFN-*γ*^+^ T cells or CD3^−^IFN-*γ*^+^ cells, CD3^+^ CD8^+^ IFN-*γ*^+^ T cells possess dual pathogenic functions: the direct cytotoxicity of effector T cells and the pro-inflammatory activity of secreted cytokines (28). This dual mechanism of direct killing and the pro-inflammatory amplification likely accounts for the enhanced platelet destruction observed in severe cases and explains why only this specific cell subset is associated with disease severity. Furthermore, no significant differences were detected in the distribution of IFN-*γ* -positive cell subsets or core immune cell populations among children with different disease courses (nITP, pITP, cITP), suggesting that abnormal activation of the CD8^+^ T cell/IFN-*γ* axis is one of the initiating factors in the onset of childhood ITP, rather than a secondary alteration that evolves dynamically with disease progression. The progression from re-diagnosis to chronicity of ITP may be associated with other factors, such as immunodeficiency of immune regulatory cells, insufficient secretion of thrombopoietin, and other complementary mechanisms (29), providing directions for further exploration of the mechanism of chronic ITP.

Correlation analysis represents a critical link between immune mechanisms and clinical phenotypes. In the current study, the proportions of conventional immune cell subsets (CD3^+^ T cells, CD4^+^ T cells, CD8^+^ T cells, and B cells) and the CD4^+^/CD8^+^ ratio showed no significant association with platelet counts or bleeding symptoms. In contrast, CD3^+^ CD8^+^ IFN-*γ*^+^ T cells were identified as the only immune indicator negatively correlated with platelet count (r = −0.2583, *P* = 0.012). This result has significant clinical implications: on the one hand, it confirmed the pathogenicity of CD3^+^ CD8^+^ IFN-*γ*^+^ T cells, as their elevated expression is directly associated with thrombocytopenia, providing key correlative evidence for the core hypothesis that the CD8^+^ T cell/IFN-*γ* axis drives disease progression in childhood ITP. It also highlights that functional activated immune cell subsets (such as IFN-*γ* -positive effector cells) are more reliable indicators of disease pathological status than the absolute proportions of naïve immune cell subsets. On the other hand, the absence of a correlation between IFN-*γ* -related immune indicators and clinical bleeding symptoms suggests that bleeding risk in pediatric ITP patients is not solely determined by platelet count but may be regulated by additional factors, including platelet function activity, vascular endothelial integrity, and coagulation factor status (30, 31). This conclusion offers a valuable reference for clinical practice: bleeding risk assessment should be individualized, integrating platelet count, immune indicators, and individual characteristics (e.g., age, comorbidities), rather than relying exclusively on single immune or laboratory parameters. Though this association reached statistical significance, the modest correlation coefficient indicates a limited linear relationship between this functional T cell subset and platelet counts, which merits cautious interpretation of its predictive utility when contextualizing biological and clinical meaning.

Several limitations of this study warrant consideration. First, this investigation employed a cross-sectional design. Although the association between the CD8^+^ T cell/IFN-*γ* axis and the clinical phenotype of childhood ITP was clarified, the causal relationship between this immune axis dysregulation and disease pathogenesis could not be revealed. Future prospective cohort studies are needed to track the dynamic changes of this immune axis and the association with disease prognosis (including recurrence rate and chronic rate), to further validate its value as a prognostic biomarker. Second, the specific molecular mechanisms underlying the effects of the CD8^+^ T cell/IFN-*γ* axis on platelet production and destruction were not fully elucidated in this study, including downstream IFN-*γ* signaling pathways and related cytokine networks. Subsequent *in vitro* functional experiments, such as cell co-culture systems and neutralizing antibody blocking experiments, are required to clarify its pathogenic pathways. Finally, the sample size of this study is relatively limited, and future research may benefit from expanding the cohort to further verify the expression characteristics of this immune axis in specific subtypes and provide more precise evidence for targeted therapy.

## 5 Conclusions

By systematically analyzing the clinical and immune characteristics of childhood ITP, this study identified the aberrant activation of the CD8^+^ T cell/IFN-*γ* axis as a core mechanism underlying disease recurrence, exacerbation, and thrombocytopenia. In addition, CD3^+^ CD8^+^ IFN-*γ*^+^ T cells were recognized as potential biomarkers for evaluating disease severity and thrombocytopenia. These findings not only enhance the current understanding of the immune pathogenesis of childhood ITP but also provide new intervention targets (such as targeted inhibition of CD8^+^ T cell activation and neutralization of IFN-*γ*) for relapsed/refractory cases, thereby facilitating the advancement of precision immunotherapy for childhood ITP.

## Data Availability

The raw data supporting the conclusions of this article will be made available by the authors, without undue reservation.
