# Correction: Correlation between the CD8^+^ T cell/IFN-*γ* axis and clinical disease progression at different phases of ITP

**DOI:** 10.3389/fped.2026.1964035

**Published:** 2026-08-24

**Authors:** Jie Ding, Chentao Shen, Jianmei Chen, Ziyang Cheng, Jianqin Li

**Affiliations:** 1 Department of Hematology and Oncology, Children's Hospital of Soochow University, Suzhou, China; 2 Suzhou Wujiang District Children's Hospital, Suzhou, China; 3 Sir Run Run Hospital, Nanjing Medical University, Nanjing, China

**Keywords:** CD8^+^ T cells, childhood ITP, immune characteristics, interferon-*γ*, therapeutic response

Figure/table caption

There was a mistake in the caption of figure [4] as published. [The symbol *** is missing in the figure 4]. The corrected caption of figure [4] appears below.

“[**P < 0.01; ***P < 0.001; ****P < 0.0001]”

The original version of this article has been updated.

