# Supplementary material for: Correlation between the CD8+ T cell/IFN-γ axis and clinical disease progression at different phases of ITP
Source: Front Pediatr. 2026 Jul 31;14:1905260. doi: 10.3389/fped.2026.1905260 (PMC13473164; doi:10.3389/fped.2026.1905260)
Supplement: Supplementary file 1 [file Table1.docx]

Supplementary Material

**Table S1. Demographic and clinical data of the study sample.**

| Variables | Patients (ITP) | | HC | |
| --- | --- | --- | --- | --- |
|  | N | % of Cases | N | % of Cases |
| Number of cases | 95 |  | 38 |  |
| Sex (*p*=0.884) |  |  |  |  |
| Female | 52 | 54.74 | 18 | 47.37 |
| Male | 43 | 45.26 | 20 | 52.63 |
| Mean age ± SD (*p*=0.207) | 68.55±44.26 months | | 78.39±29.14 months | |
| Range of age | 3-196 months | | 17-132 months | |
| Relapse status |  |  |  |  |
| Newly diagnosed ITP | 56 | 58.95 |  |  |
| Relapsed ITP | 39 | 41.05 |  |  |
| Disease phases |  |  |  |  |
| nITP | 44 | 46.31 |  |  |
| pITP | 21 | 22.11 |  |  |
| cITP | 30 | 31.58 |  |  |
| Disease severity grading |  |  |  |  |
| Severe ITP | 15 | 15.79 |  |  |
| Non-severe ITP | 80 | 84.21 |  |  |
